# Maintenance of Glia in the Optic Lamina Is Mediated by EGFR Signaling by Photoreceptors in Adult Drosophila

**DOI:** 10.1371/journal.pgen.1005187

**Published:** 2015-04-24

**Authors:** Yuan-Ming Lee, Y. Henry Sun

**Affiliations:** 1 Institute of Molecular Biology, Academia Sinica, Taipei, Taiwan; 2 Institute of Genomic Sciences, National Yang-Ming University, Taipei, Taiwan; New York University, UNITED STATES

## Abstract

The late onset of neurodegeneration in humans indicates that the survival and function of cells in the nervous system must be maintained throughout adulthood. In the optic lamina of the adult *Drosophila*, the photoreceptor axons are surrounded by multiple types of glia. We demonstrated that the adult photoreceptors actively contribute to glia maintenance in their target field within the optic lamina. This effect is dependent on the epidermal growth factor receptor (EGFR) ligands produced by the R1-6 photoreceptors and transported to the optic lamina to act on EGFR in the lamina glia. EGFR signaling is necessary and sufficient to act in a cell-autonomous manner in the lamina glia. Our results suggest that EGFR signaling is required for the trafficking of the autophagosome/endosome to the lysosome. The loss of EGFR signaling results in cell degeneration most likely because of the accumulation of autophagosomes. Our findings provide *in vivo* evidence for the role of adult neurons in the maintenance of glia and a novel role for EGFR signaling in the autophagic flux.

## Author Summary

Degeneration of the nervous system can be viewed as a failure to maintain cell survival or function in the nervous system. The late onset of neurodegeneration in humans indicates that the cell survival in the nervous system must be maintained throughout our lives. Neuronal survival is maintained by neurotrophic factors in adults; however, it is unclear whether glia survival is also maintained throughout adulthood. Here, we use the *Drosophila* visual system as a model to address the role played by adult neurons for the active maintenance of glia. We demonstrated that the adult photoreceptors secrete a signaling molecule, which is transported to the brain to act on the lamina glia and maintain its integrity. When this signaling pathway is blocked, the lamina glia undergoes a progressive and irreversible degeneration. The primary defect occurs in the trafficking from the late endosome and autophagosome to the lysosome. This defect leads to an accumulation of autophagosomes and subsequent cell degeneration as a result of autophagy. Our findings provide *in vivo* evidence for a novel aspect of the neuron-glia interaction and a novel role for EGFR signaling in regulating the maintenance and degeneration of the nervous system.

## Introduction

The degeneration of the nervous system can be viewed as a failure to maintain cell survival and function within the nervous system. In mammals, the survival of neurons during development and adulthood is actively maintained by the neurotrophic factors produced by other neurons or glias [1, 2]. In *Drosophila*, neurotrophin-like proteins are secreted by neuron, muscles, and glia to maintain the survival of specific subsets of neurons during development [3–6].

The survival of glia during development can be reciprocally dependent on the trophic support from neurons. For example, in mammals, the neuregulin NRG1, neurotrophins, transforming growth factor alpha (TGFα), and purines can act on various types of glia to maintain their survival [7–10]. In the *Drosophila* embryonic central nervous system (CNS), the survival of the longitudinal glia (LG) and midline glia (MG) are dependent on the neuregulin-like epidermal growth factor receptor (EGFR) ligands Vein (Vn) and Spitz (Spi), respectively [6, 11, 12]. The PVR ligand PVF1 is also required for MG survival [13]. However, it is unclear whether glia survival is actively maintained in adult flies.

We hypothesized that glia survival is actively maintained in the adult visual system via the gliotrophic factors secreted by the closely associated cells. Because endocytosis, which is involved in the internalization of many activated receptors, strongly affects cellular signaling outcomes [14, 15], blocking endocytosis should perturb these signaling events. Therefore, we expressed temperature-sensitive Shibire (Shi^ts1^), driven by the *repo-GAL4*, which is expressed in most glia [16]. The *shi* gene is the fly homolog of mammalian *dynamin* [17], which is required for multiple forms of endocytosis [18–20], as well as vesicle recycling, which indirectly affects exocytosis [21]. Shi^ts1^ is dominant-negative at non-permissive temperatures, which thereby blocks endocytosis [19]. The use of this approach in the fly visual system enabled us to examine the gliotrophic requirements during the adult stage and precisely determine the specific cell types involved.

EGFR signaling is highly conserved evolutionarily and is involved in many developmental processes [22, 23] and pathological conditions in vertebrates [24–26]. The ligand-bound EGFR can be internalized by endocytosis. In the endosome, the EGFR can either recycle back to the cell surface or undergo lysosomal degradation [27]. The activated EGFR can signal from the cell surface and continues to signal from the early endosome before it is eventually ubiquitinated and degraded in the lysosome [28–30]. Five EGFR ligands exist in *Drosophila*: four agonists (Spi, Keren (Krn), Gurken (Grk) and Vn) and one antagonist (Argos) [22]. During eye development, EGFR signaling, which is mediated by Spi and Krn, drives the progressive differentiation of multiple retinal cell types [31]. Spi is subsequently expressed in the photoreceptors and transported to the axon termini in the lamina to regulate EGFR on the lamina neurons and the differentiation of cartridge neurons [32]. The regulation and function of the EGFR ligands sent through the photoreceptor axon to their target field during eye development is well characterized [22, 32–37]. However, the role of the EGFR ligands in the adult visual system has not been studied. Spi and Vn exert a gliotrophic function for glia in the embryonic CNS [6, 11, 12]; thus, we investigated whether EGFR signaling is also important in the adult visual system.

Tissue degeneration may be a result of excessive cell death. The EGFR/Ras/Raf/MAPK signaling pathway can protect cells from apoptosis via direct inhibition of the pro-apoptotic protein Hid [38, 39]. The ligand-activated EGFR can bind to the autophagy protein Beclin-1 [40] and suppress autophagy in mammals [41]. Therefore, the loss of EGFR signaling can cause either apoptosis or autophagy, which most likely depends on the cell type and cellular context [42]. We demonstrated that the adult R1-6 photoreceptor-secreted Spi acts on the lamina glia EGFR to maintain glial integrity. In the absence of the EGFR signaling, the lamina glia undergoes degeneration. Our results suggest that the primary defect caused by a lack of EGFR signaling is not apoptosis but the accumulation of autophagosomes, which subsequently leads to cell degeneration without cell loss. Therefore, our results demonstrate that the adult photoreceptors actively maintain the functional integrity of the glia in their target field. In addition, our findings indicate a novel role for EGFR signaling in the promotion of late endosome/autophagosome trafficking to lysosomes and identify a novel form of degeneration that does not involve cell loss.

## Results

### Dynamin function blockade caused a cell-autonomous degeneration of the lamina glia

**Fig 1:**
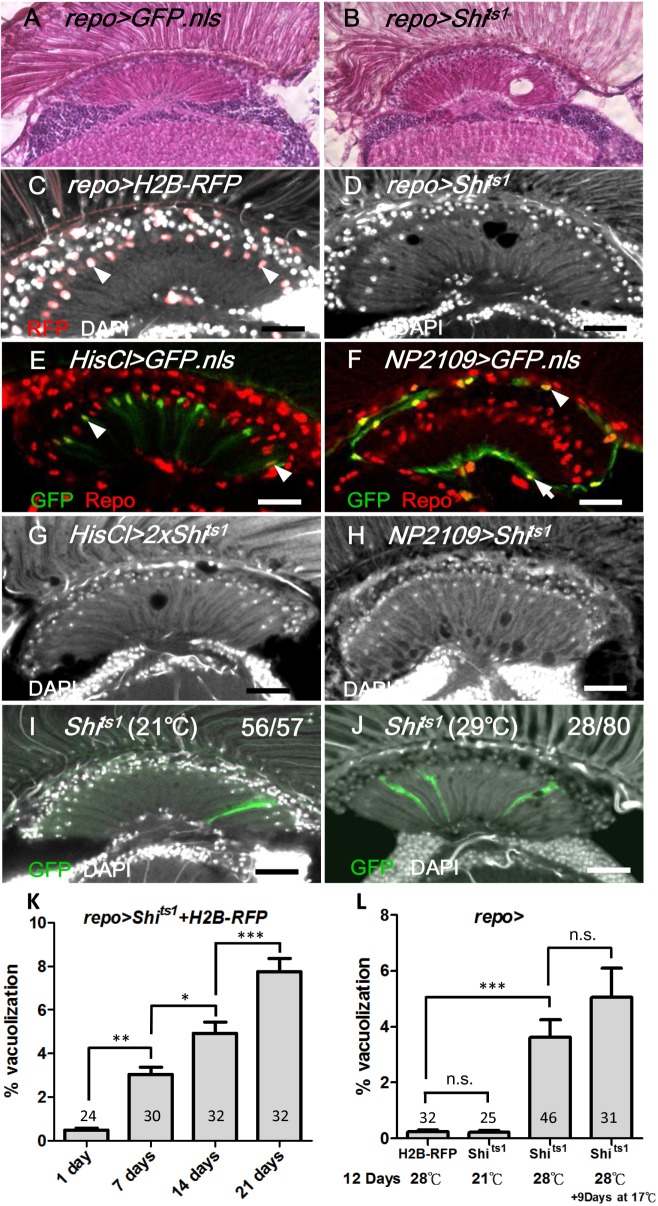
Shi^ts^ induced cell-autonomous glia degeneration in optic lamina. H&E staining of adult head sections of (A) *repo>GFP*.*nls* and (B) *repo>Shi* ^*ts1*^ at 29°C for 3 days. Lamina degeneration was identified as vacuoles in (B). Cryosection of adult (C) *repo>H2B-RFP* exhibited the expression of the nuclear red fluorescent protein (RFP) in the glia (epithelial glial nucleus: arrowhead) and (D) *repo>Shi* ^*ts1*^ at 28°C for 12 days. Vacuoles were identified in the lamina neuropile (D). Epithelial (arrowhead) (E), marginal (arrow) and distal satellite (arrowhead) glia nuclei (F) are labeled by *HisCl-GAL*4 and *NP2109-GAL4*, respectively. Note that *HisCl-GAL4* is not expressed in all epithelial glia. (G, H) Weak lamina vacuolization was identified in *HisCl>Shi* ^*ts1*^ (G, 0.92%) and *NP2109>Shi* ^*ts1*^ (H, 1.46%) at 29°C for 14 days. (I, J) A single MARCM glia clone (GFP, green) expressed Shi^ts1^ at 21°C (I) and 29°C (J). (J) A vacuole occurred within a glial clone. DAPI (white) stains the nuclei (C, D, and G-J). (K) The percentage of the vacuole area in the lamina of the *repo>Shi* ^*ts1*^ flies at 28°C progressively increased. n indicated in each column. *P*-values were calculated using one-way ANOVA with Bonferroni’s post-test. (L) When *repo>Shi* ^*ts1*^ flies were shifted to 28°C for 12 days and then shifted to 17°C for 9 additional days, the vacuolization was not alleviated. *P*-values were calculated using one-way ANOVA with Tukey’s post-test. n indicated in each column.

We inhibited the endocytic function specifically in the glia of adult flies using a targeted expression of Shi^ts1^, which was driven by the glia-expressing *repo-GAL4* (abbreviated *repo>Shi* ^*ts1*^). At the non-permissive temperature, the *repo>GFP*.*nls* and *repo>H2B-RFP* flies exhibited normal retina and optic lobe structures (Fig 1A and 1C). The lamina in the *repo>Shi* ^*ts1*^ adults were normal when cultured at the permissive temperature (21°C) (Fig 1L); however, they exhibited vacuoles in the optic lamina two days after a shift to the non-permissive temperature (28°C) (Fig 1B and 1D). The phenotype progressively worsened, and 5% of the lamina volume became vacuolated at 14 days (Fig 1K). When the *repo>Shi* ^*ts1*^ flies were shifted to 28°C for 12 days and then shifted to 17°C for 9 days, the vacuolization phenotype was not reversed (Fig 1L). Thus, blocking Shi function in the glia causes an irreversible and progressive degeneration of the optic lamina.

**Fig 2:**
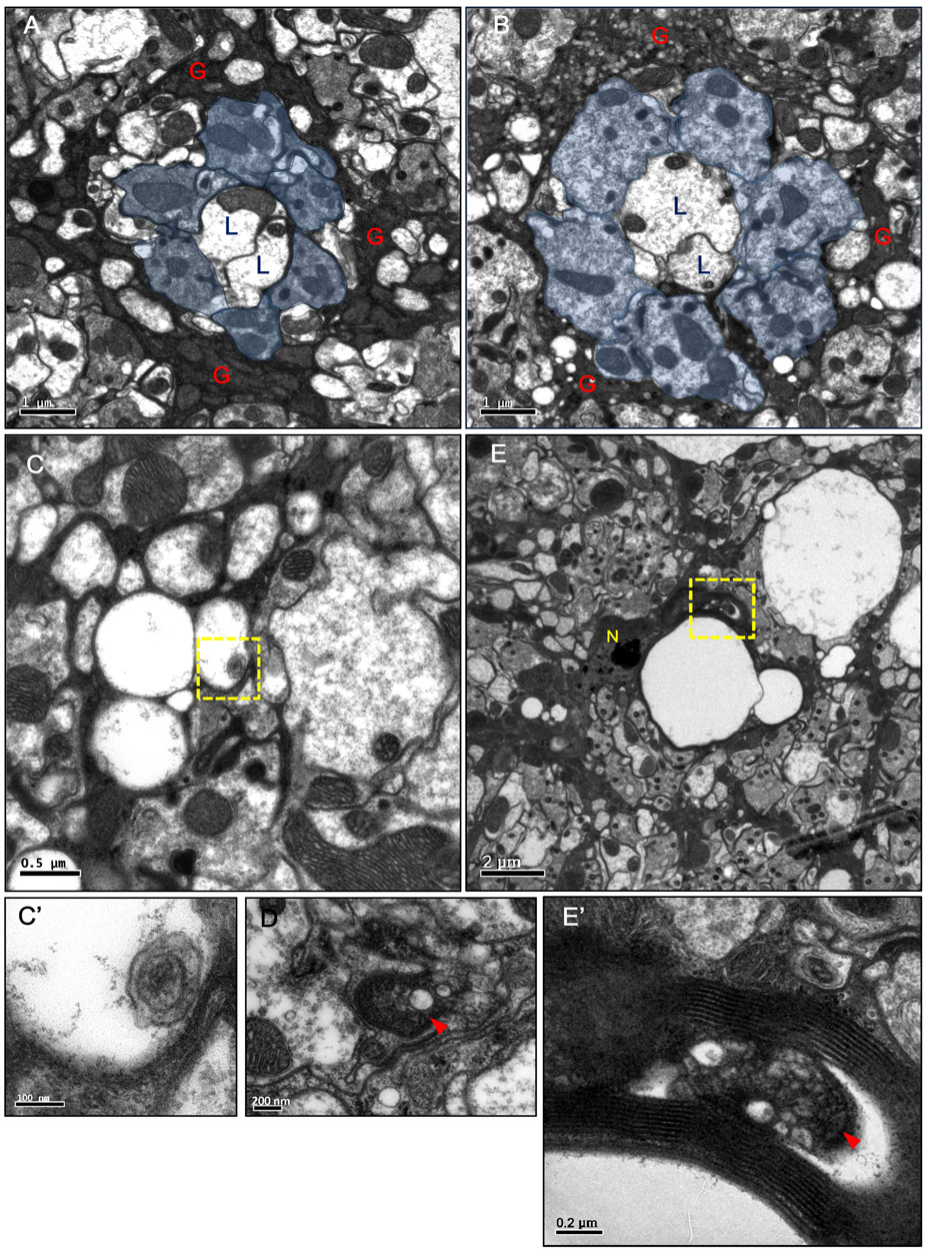
Vacuoles and autophagosome-like structures in the degenerating epithelial glia. (A-E) Horizontal sections of the adult head lamina cartridge. (A) In *repo>GFP*.*nls* flies, two lamina neurons L1, L2 axons (L) and R1-6 axons (blue area) were surrounded by the electron-dense cytoplasm of the epithelial glia (G). The section was examined at three different depths, and the size of a single cartridge is not significantly different at different depths. (B-D) *repo>Shi* ^*ts1*^ adults maintained at 29°C for 4 days. Axons were enlarged but intact. A large number of small and medium vacuoles were identified in the epithelial glia. (C) Small vacuoles in the glia that contained a double-membrane structure (arrowhead). (C’) Higher magnification of the boxed area in (C). (D) A double-membrane autophagosome-like structure (arrowhead) in the glial cytoplasm. (E) In *repo* ^*ts*^ *>DER* ^*DN*^ adults maintained at 29°C for 2 days, large vacuoles were identified in between the lamina cartridges. N: glial nuclei. (E’) Higher magnification of the boxed area in (E), which shows autophagosome-like vesicles (arrowhead) in the glia cytoplasm.

We next examined the specific cell types that were affected by vacuolization. The optic lamina possesses six distinct glia cell types, namely, fenestrated glia, distal satellite glia, proximal satellite glia, epithelial glia, marginal glia, and chiasm glia [43]. The location of the vacuoles correlated with the location of the epithelial glia and, to a lesser extent, the marginal glia. Shi^ts1^ expression driven by an epithelial glia-specific *HisCl-Gal4* (Fig 1E) or a marginal glia-specific *NP2109-Gal4* (Fig 1F) also caused a weak lamina vacuolization (Fig 1G and 1H). We used the MARCM method [44] to clonally express Shi^ts1^ and GFP in glial cells. At 21°C, the MARCM clones did not exhibit defects (Fig 1I). At 29°C, of 70 MARCM clones, 28 clones exhibited vacuoles, which can be detected within a single cell clone (Fig 1J). We further examined the phenotype using electron microscopy (EM). In the wild type adult optic lamina, one lamina cartridge contains five lamina neurons, with the L1/L2 terminals in the center, surrounded by six photoreceptor terminals, which are then surrounded by epithelial glia (Fig 2A). In the *repo>Shi* ^*ts1*^ lamina, small and large vacuoles were identified within the electron-dense glial cytoplasm, and the R cell axons were enlarged but contained no vacuole (Fig 2B). Most vacuoles appeared empty, with only a few vacuoles that contained double membrane structures (Fig 2C and 2C’). We also observed double membrane autophagosome-like structures [45] within the cytoplasm (Fig 2D). These results suggest that blocking Shi function in the lamina glia caused a cell-autonomous vacuolization.

The neural response to a light pulse was measured by electroretinogram (ERG), which is composed of an “ON” transient, a depolarization, and an “OFF” transient (S1A Fig). The depolarization measures the transmission within the photoreceptor axons, whereas the ON and OFF transients measure the synaptic transmission from the photoreceptor neurons to the lamina neurons [46, 47]. We demonstrated that the *repo>Shi* ^*ts1*^ flies exhibit a normal depolarization but a loss of the ON and OFF transients on the ERG on day 3 (S1B–S1D Fig). This result suggests that while the neural transmission along the photoreceptor axon is normal, the synaptic transmission from the photoreceptor neurons to the lamina neurons is defective. Because the lamina synaptic region is wrapped by epithelial glia, which is known to recycle the neurotransmitters from the photoreceptors [48–50], the synaptic transmission defect is most likely a result of an epithelial glia dysfunction.

### R1-6 photoreceptors are required for lamina glia maintenance

**Fig 3:**
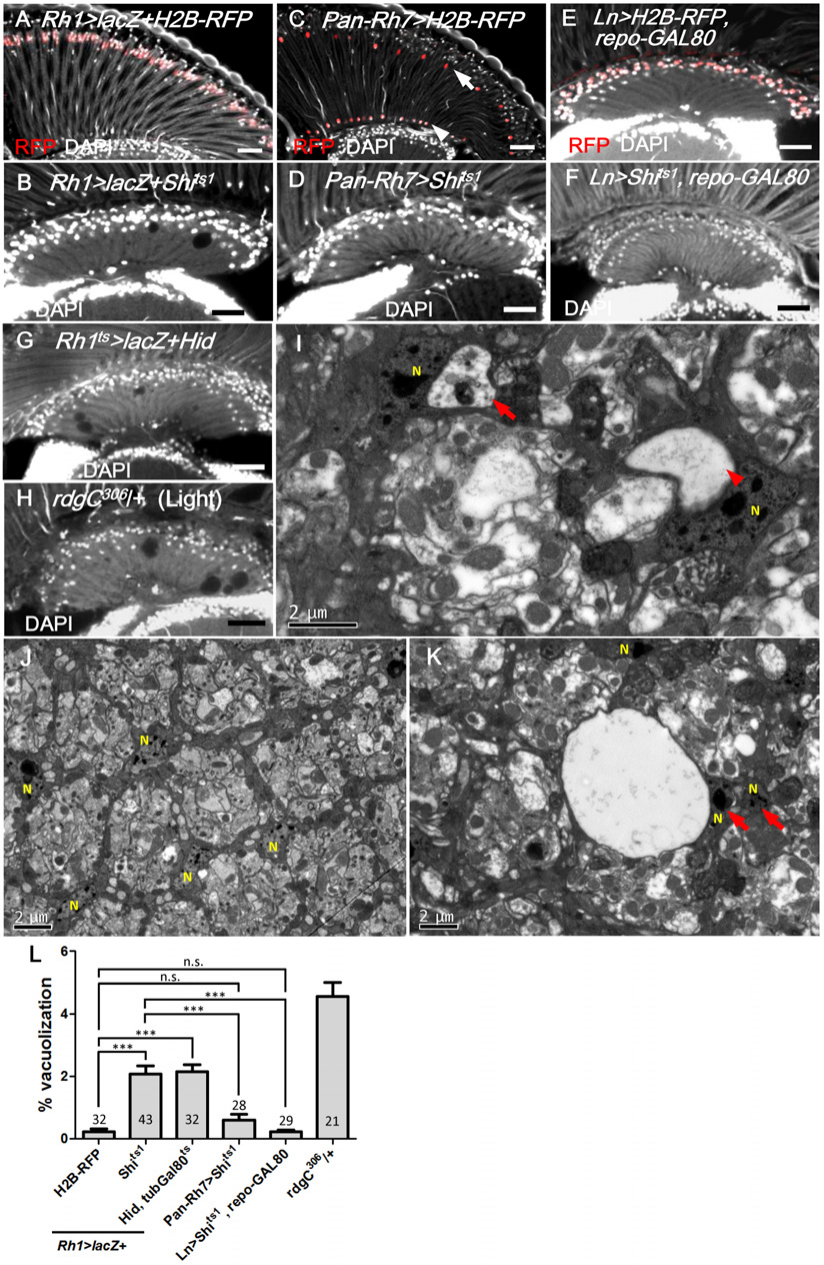
R1-6 photoreceptors are required for lamina glia maintenance. (A) *Rh1>lacZ+H2B-RFP* exhibited nuclear RFP expression in the R1-6 photoreceptors (red). (B) *Rh1>lacZ+Sh* ^*its1*^ maintained at 29°C for 14 days resulted in lamina vacuolization. (C) *Pan-Rh7>H2B-RFP* exhibited expression in R7 (arrow) and R8 (arrowhead) (red). (D) *Pan-Rh7>Shi* ^*ts1*^ incubated at 29°C for 14 days did not exhibit lamina degeneration. (E) Lamina monopolar (L) neurons (red) were selectively labeled by the *Ln-GAL4* driven *H2B-RFP* with *repo-GAL8*0. (F) *Ln>Shi* ^*ts1*^, *repoGAL80* shifted to 29°C for 14 days did not exhibit lamina degeneration. (G) R1-6 photoreceptors were killed in *Rh1* ^*ts*^ *>lacZ+Hid* shifted to 29°C for 14 days. *Rh1* ^*ts*^ indicates *tubGAL80* ^*ts*^; *Rh1-GAL4*. Degeneration was induced in the lamina in addition to the retina. (H) *rdgC* ^*306*^/+ mutant exposed to constant light for 14 days exhibited degeneration in the retina, as well as the lamina. DAPI: nuclei (white in A-H). (I, K) *Rh1>lacZ+Shi* ^*ts1*^ at 29°C for 4 days exhibited vacuoles in the electron dense cytoplasm of epithelial glia or near the glia nucleus. Arrow: vacuole with internal debris. Arrowhead: large vacuole. (J) Lamina cartridge of *Rh1>lacZ+H2B-RFP*. N indicates glial nuclei. (L) The percentage of the vacuole area in the lamina at 29°C for 14 days was examined. All *P*-values were calculated using one-way ANOVA with Tukey’s post-test. Scale bar: 20 μm.

Because endocytosis is involved in many signaling pathways in the receiving cells, the lamina glia may receive a gliotrophic signal via endocytosis. One potential source for the gliotrophic factor may be the photoreceptors, since their axons form synaptic contacts with both the monopolar lamina neurons and the epithelial glia in the lamina cartridge [51]. We demonstrated that the expression of Shi^ts1^ using a R1-6 photoreceptor-specific *Rh1-GAL4* (Fig 3A) caused a lamina vacuolization (Fig 3B and 3L) similar to the *repo>Shi* ^*ts1*^ flies. Dynamin is also required for vesicle recycling [21]; thus, the loss of Shi function could affect the vesicle recycling, which leads to the loss of ligand secretion, as demonstrated for Wg secretion [52]. In the *Rh1>Shi* ^*ts1*^ flies, the structure of the lamina cartridge of the photoreceptor axons was disorganized, and the lamina neuropile contained vacuoles in the epithelial glia layer (Fig 3I and 3K). In the glial nuclei layer, the vacuoles formed near the nuclei (Fig 3I). A glial nucleus is squeezed by a large vacuole to become adjacent to another glial nucleus (arrow in Fig 3K compared with 3J). When the expression was driven by the R7/8-specific *Pan-Rh7-Gal4* (Fig 3C), no lamina vacuolization was identified (Fig 3D and 3L). A specific lamina L2-5 neuron *Ln-GAL4-*driven expression, combined with a *repo-GAL80* to block the *Ln-GAL4* activity in the satellite glia (Fig 3E), did not cause lamina vacuolization (Fig 3F and 3L). Furthermore, when the R1-6 photoreceptors were killed via the expression of the apoptotic gene *hid*, lamina vacuolization was induced (Fig 3G and 3L). We also ablated the photoreceptors in a different manner. The rhodopsin protein phosphatase RdgC is expressed in the retina and ocelli, and the *rdgC* ^*306*^ mutant exhibits normal lamina morphology at birth but a light-dependent retinal degeneration [53, 54]. The *rdgC* ^*306*^ mutant exhibited degeneration in the lamina and retina after constant illumination for 14 days (Fig 3H and 3L). These results indicate that R1-6 photoreceptors are required for lamina glia vacuolization.

### EGFR signaling in the lamina glia is required and sufficient to autonomously maintain the glia

**Fig 4:**
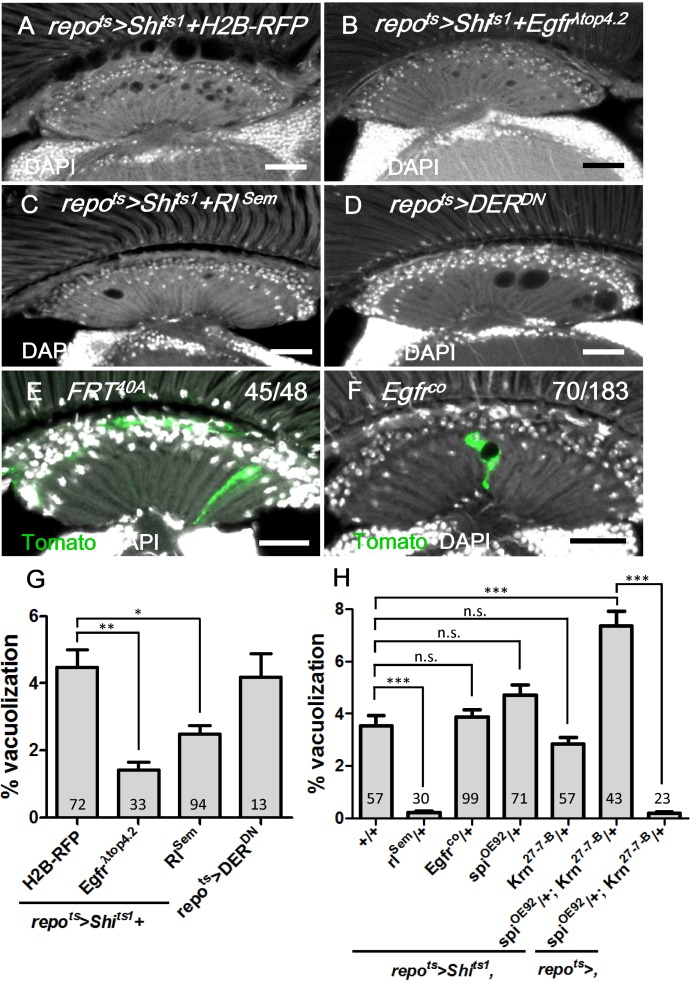
EGFR is required and sufficient in the lamina glia to maintain glia integrity. (A) *repo* ^*ts*^ *>Shi* ^*ts1*^ *+H2B-RFP*, (B) *repo* ^*ts*^ *>Shi* ^*ts1*^ *+Egfr* ^*λtop*^ and (C) *repo* ^*ts*^ *>Shi* ^*ts1*^ *+Rl* ^*Sem*^ incubated for 12 days at 28°C. (D) *repo* ^*ts*^ *>DER* ^*DN*^ for 7 days at 28°C. (E) Epithelial glia MARCM clone and (F) *Egfr* ^*co*^ mutant glia MARCM clone (labeled with Tomato, green). The penetrance is indicated as the number of samples with vacuoles over the total number of samples. DAPI: nuclei (white in A-F). (G) Percentage of vacuole area in the lamina neuropile of (A-D). The *P*-values were calculated using one-way ANOVA with Dunnett’s post-test. (H) Percentage of vacuole area in the lamina of *repo* ^*ts*^ *>Shi* ^*ts1*^ flies in the indicated genetic background. The adults were shifted to 28°C for 12 days. The *P*-values were calculated using one-way ANOVA with Tukey’s post-test. Scale bar: 20 μm.

EGFR, which is internalized by endocytosis and continues to signal from the early endosome, is required for glia survival in the embryonic CNS [6, 11, 12]; thus, we investigated whether EGFR signaling acts in the adult lamina glia to maintain the glia. To specifically drive expression in adult glia, we combined the *repo-GAL4* with *tub-Gal80* ^*ts*^ (abbreviated as *repo* ^*ts*^). In these flies, GAL4 activity is suppressed by the GAL80^ts^ at the permissive temperature, and a shift to the non-permissive temperature after eclosion induces GAL4 activity. The coexpression of a constitutively active form of EGFR (*repo* ^*ts*^ *>Shi* ^*ts1*^ *+Egfr* ^*λtop4*.*2*^) suppressed the *repo* ^*ts*^ *>Shi* ^*ts1*^ vacuolization phenotype (Fig 4A and 4B and 4G). The phenotype could also be rescued via the coexpression of an active form of the fly MAPK Rolled (*repo* ^*ts*^ *>Shi* ^*ts1*^ *+Rl* ^*Sem*^) (Fig 4C and 4G) or a heterozygotic combination with the gain-of-function allele *rl* ^*Sem*^ (Fig 4H). These results suggest that EGFR/MAPK signaling is sufficient to maintain glial integrity, and the vacuolization phenotype was not a result of the EGFR trapped at the cell surface, but rather a loss of signaling. Conversely, the expression of a dominant-negative *Drosophila* EGFR (DER^DN^) in the glia (*repo* ^*ts*^ *>DER* ^*DN*^) caused a similar lamina vacuolization as in the *repo* ^*ts*^ *>Shi* ^*ts1*^ flies (Fig 4D and 4G). In the *Egfr* ^*co*^ mutant flies, vacuoles could be identified within the clones (70/183 in Fig 4F compared with 45/48 in Fig 4E). These results suggest that EGFR signaling is cell-autonomously required in the lamina glia to maintain their integrity.

### EGFR ligands from the retina are required for lamina glia maintenance

**Fig 5:**
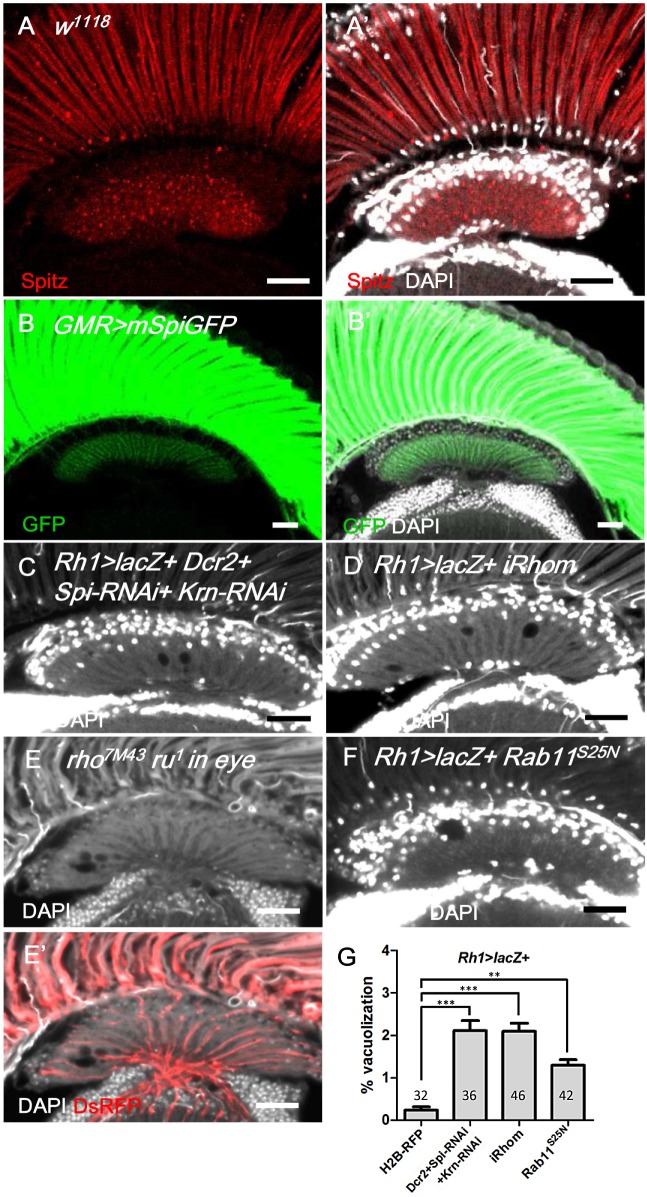
Spitz from photoreceptors is transported to the lamina and is required for lamina glia maintenance. (A, A’) Anti-Spitz (red) immunostaining of *w* ^*1118*^ adult head. Spitz can be detected in the retina and lamina. (B, B’) The full-length transmembrane form of Spitz-GFP (mSpi, green) expressed in the retina in *GMR>mSpiGFP* flies was predominantly identified in the photoreceptor soma and terminally localized in the lamina cartridge. mSpitz-GFP requires processing by Rho and Star to become a secreted form. Overexpressed mSpitzGFP has been demonstrated to be retained in the perinuclear ER even in the presence of endogenous Rho/Star [56] (C) Knockdown of both EGFR ligands Spi and Krn in R1-6 photoreceptors in *Rh1>Dcr2+Spi-RNAi+Krn-RNAi* and (D) blockade of Spi processing in *Rh1>lacZ+iRhom* exhibited lamina degeneration after shifting to 29°C for 14 days. (E-E’) Lamina degeneration in whole eye *rho* ^*7M43*^ *ru* ^*1*^ double mutant clones at 28°C for 12 days. The clone is labeled by DsRed (red). (F) Spi secretion is inhibited in *Rh1>lacZ+Rab11* ^*S25N*^. (G) The percentages of the vacuole areas of (C, D, F) in lamina at 29°C for 14 days were examined. All *P*-values were calculated using one-way ANOVA with Tukey’s post-test. DAPI: nuclei (white in A’, B’, C-F). Scale bar: 20 μm.

What is the gliotrophic signal produced by the photoreceptors? Based on RNA microarray data, *spitz*, *Keren*, and *vein*, but not *gurken*, are expressed in the adult eye [55]. The Spi protein can be predominantly detected in the adult retina and as puncta in the lamina (Fig 5A). The targeted expression of full length Spi (mSpi-GFP) [56] in photoreceptors (*GMR>mSpi-GFP*) exhibited a strong GFP signal in the retina and a weak signal in the lamina neuropile, where the photoreceptor axons terminate (Fig 5B). These results indicate that Spitz expressed from the photoreceptors can be transported from the retina to the lamina. The knockdown of both Spi and Krn in the photoreceptor cells also caused lamina vacuolization (Fig 5C and 5G). Although the severity of the *repo* ^*ts*^ *>Shi* ^*ts1*^ fly phenotype was not affected by a reduction in the dosage of *Egfr*, *spi* or *Krn*, it was strongly enhanced in *spi* and *Krn* double-heterozygous mutants (Fig 4H). These results suggest that the EGFR ligands Spi and Krn are redundantly required in the photoreceptors to prevent lamina vacuolization.

The EGFR ligands Spi, Krn and Grk are synthesized as membrane-bound precursors and must be transported by the chaperone Star and cleaved in the ER by the intramembranous protease Rhomboid (Rhom) to acquire their active secreted form [34]. We generated whole-eye *rho* ^*7M43*^ *ru* ^*1*^ clones that have double null mutations for *rhom-1* (*rho*) and *rhom-3* (also referred to as *roughoid*, *ru*) [57] and identified lamina vacuolization in these mutants (Fig 5E). iRhom is an inactive Rhomboid-like pseudoprotease that promotes the degradation of EGFR ligands in the ER [58]. We expressed iRhom in the retina to promote the degradation of EGFR ligands in the signal-producing cells. Lamina vacuolization was identified in the *Rh1>iRhom* flies (Fig 5D and 5G). Rab11 is required for Spitz secretion in the larval photoreceptors [35]. The expression of a dominant-negative Rab11^S25N^ in the R1-6 photoreceptors caused a mild lamina vacuolization (Fig 5F and 5G). These data indicate that the transport, processing and secretion of the EGFR ligands is required in the R1-6 photoreceptors to maintain lamina glial integrity, which suggests that the R1-6 photoreceptor neurons are the source of EGFR ligands.

### EGFR signaling in the lamina glia is dependent on the EGFR ligands from the R1-6 photoreceptors

**Fig 6:**
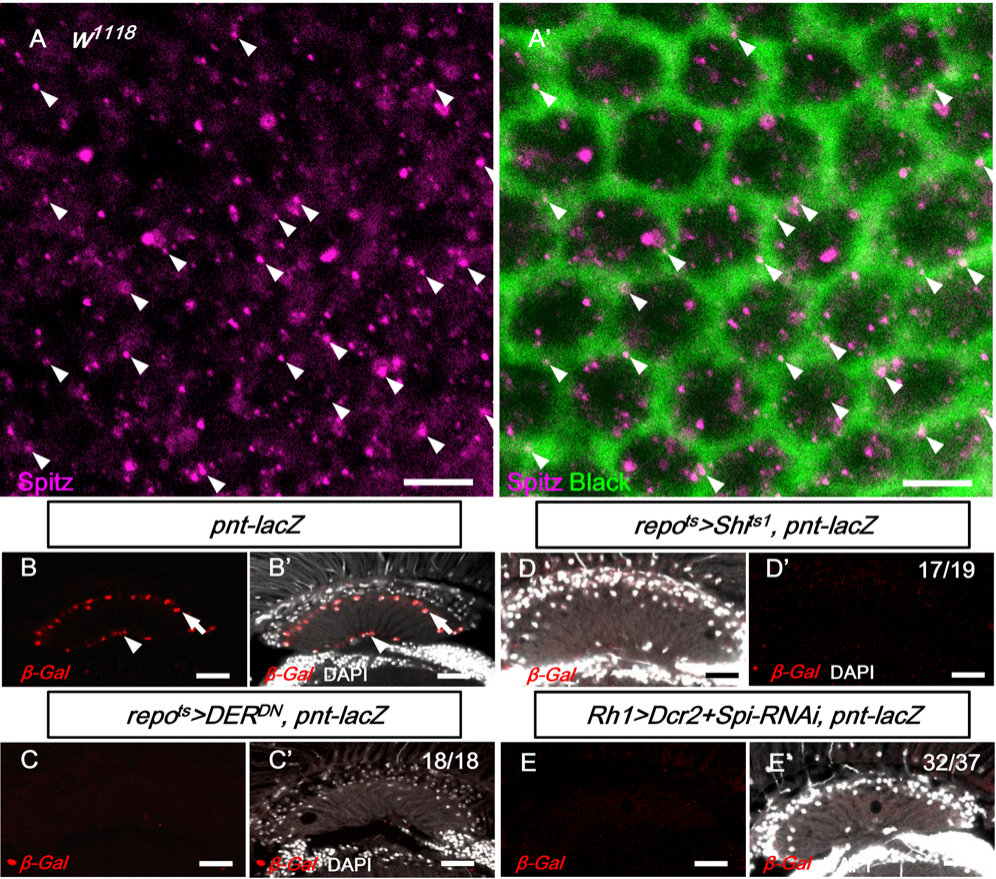
EGFR signaling in the lamina glia is dependent on Spitz from the R1-6 photoreceptors. (A, A’) Anti-Spitz detected Spitz (magenta) colocalizing (arrowhead) with epithelial glia cytoplasm marked by anti-Black (green). Scale bar: 5 μm. (B-B’) EGFR reporter *pointed-lacZ* (*pnt-lacZ)* exhibited expression in the epithelial (arrow) and marginal glia (arrowhead). (C, C’) Dominant-negative form of EGFR (DER^DN^) expressed in glia at 28°C for 3 days inhibited *pnt-lacZ* expression. (D, D’) *pnt-laZ* expression was lost in Shi^ts^-expressing glia at 28°C for 3 days. (E, E’) The knockdown of Spi in R1-6 photoreceptors at 28°C for 12 days also inhibited *pnt-lacZ* expression in the glia. The penetrances of (B-E) are shown in the upper right corner of each panel. DAPI: nuclei (white in B’-E’). Scale bar: 20 μm.

The previous results suggested that the EGFR ligand Spi secreted by the photoreceptors can be transported to the lamina and activate EGFR in the lamina glia. Spi can be found in the photoreceptor axons in the lamina and colocalizes, in part, with Black, an aspartate decarboxylase specific for the cytoplasm of epithelial glia cells [50] (Fig 6A). This result suggests that Spitz can be secreted from the photoreceptor axons and internalized in the epithelial glia. The EGFR target *pointed-lacZ* can be used as a reporter for EGFR signaling [59–61] and was expressed in the lamina epithelial and marginal glial cells (Fig 6B). The *pnt-lacZ* expression in the lamina was lost after the blockade of EGFR signaling (in *repo* ^*ts*^ *>DER* ^*DN*^; Fig 6C) or endocytosis (*repo* ^*ts*^ *>Shi* ^*ts1*^; Fig 6D) in the glia. When the Spitz expression was knocked down in the R1-6 photoreceptors (in *Rh1>Dcr2+Spi-RNAi*), the *pnt-lacZ* expression was lost in the lamina glia (Fig 6E). These results demonstrate that EGFR signaling is active in the lamina glia and is dependent on Spi produced by the R1-6 photoreceptors.

### Lamina glia vacuolization is partially a result of autophagy

**Fig 7:**
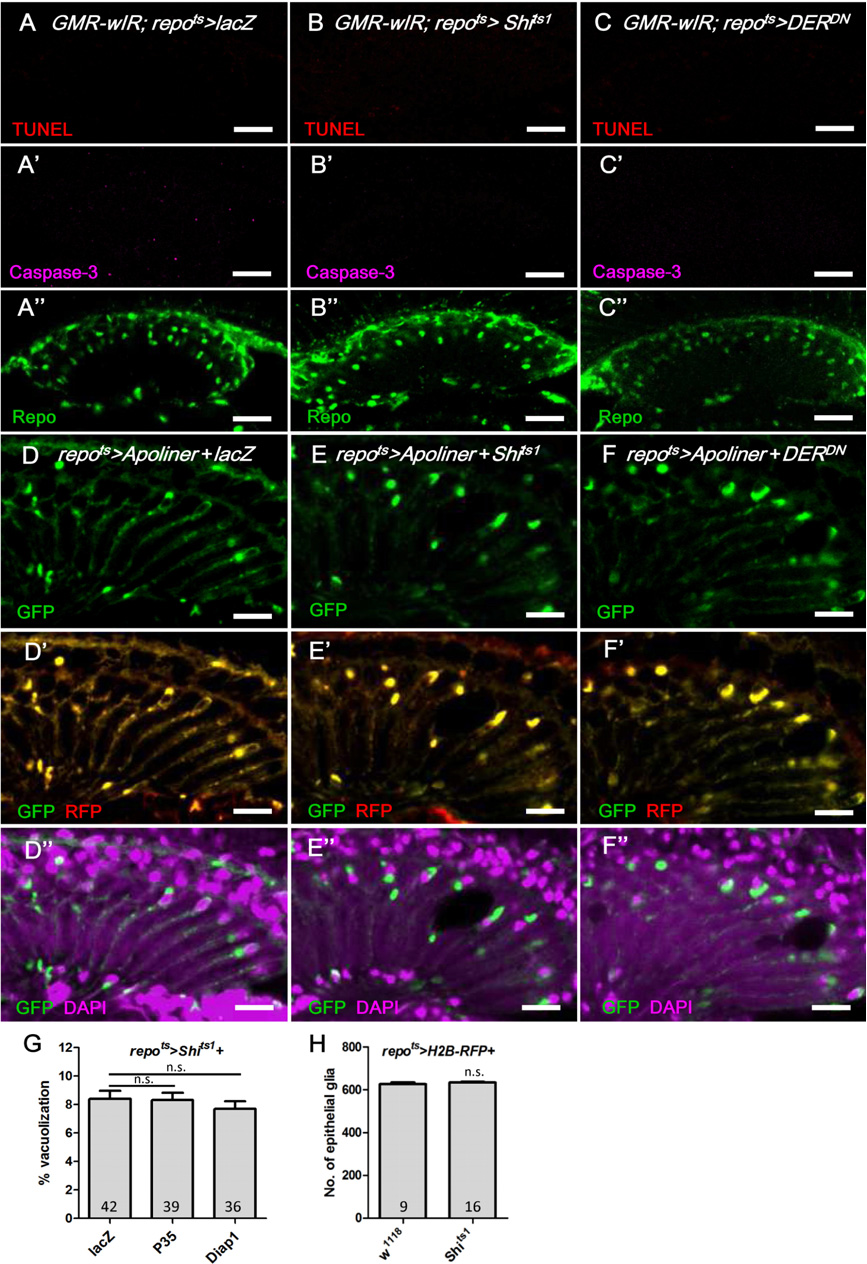
Apoptosis is not involved in the epithelial glia degeneration mediated by blockade of EGFR signaling. (A-C) Immunostaining of TUNEL assay (red), active Caspase-3 (magenta, A’-C’) and Repo (green, A”-C”). (A) *GMR-wIR; repo* ^*ts*^ *>lacZ*. (B) *GMR-wIR; repo* ^*ts*^ *>Shi* ^*ts1*^. (C) *GMR-wIR; repo* ^*ts*^ *>DER* ^*DN*^. *GMR-wIR* was used to reduce the autofluorescence of the eye pigments. Scale bar: 20 μm. (D-F) The *in vivo* fluorescent sensor of caspase activity (Apoliner) indicated there was no active caspase activity in the glia. (D) *repo* ^*ts*^ *>Apoliner+lacZ*. (E) *repo* ^*ts*^ *>Apoliner+Shi* ^*ts1*^. (F) *repo* ^*ts*^ *>Apoliner+DER* ^*DN*^. All adults were shifted to 28°C for 5 days. nls-GFP (green, D-F); merge of GFP (green) and mRFP (red) in (D’-F’); merge of nls-GFP and DAPI (magenta) in (D”-F”). Scale bar: 10 μm. (G) Percentage of vacuole area in the lamina in *repo* ^*ts*^ *>Shi* ^*ts1*^ flies that coexpressed the anti-apoptotic factors P35 and Diap1. The adults were shifted to 28°C for 21 days. The *P*-values were calculated using one-way ANOVA with Tukey’s post-test. (H) The cell number of the epithelial glia was not reduced in the vacuolated lamina. The numbers of epithelial glia in the lamina of *repo>H2B-RFP* and *repo>H2B-RFP+Shi* ^*ts1*^ female adults incubated at 28°C for 14 days were examined by counting the nuclear RFP at the epithelial layer from the entire Z-stacks of confocal images. *P*-values were calculated via Mann-Whitney tests.

We next addressed the cellular basis of lamina glia vacuolization. Apoptosis, which was assessed by activated Caspase-3 and TUNEL assays, was not identified in the *repo* ^*ts*^ *>Shi* ^*ts1*^ and *repo* ^*ts*^ *>DER* ^*DN*^ flies (Fig 7A–7C) compared with the control experiments (S2A and S2B Fig). We also used Apoliner, which is an *in vivo* fluorescent sensor for activated caspases [62] that contains a caspase cleavage site flanked by a membrane-targeted RFP and a nuclear-targeted GFP. The nuclear GFP is typically retained at the cell membrane by tethering to the mRFP (Fig 7D); however, it relocalizes into the nucleus following the caspase site cleavage. The coexpression of Apoliner with Shi^ts1^ or DER^DN^ resulted in a perinuclear distribution of the GFP that was colocalized with mRFP (Fig 7E and 7F), which indicates that Caspase-3 was not activated. The *repo* ^*ts*^ *>Shi* ^*ts1*^ vacuolization phenotype was not rescued by the coexpression of the anti-apoptotic proteins P35 [63] or Diap1 [64–66], even at 21 days (Fig 7G). Consequently, the vacuolization is most likely not a result of apoptosis. The vacuolization does not involve a reduction in the number of epithelial glia cells, as demonstrated by the nuclear RFP signal in the *repo>Shi* ^*ts1*^ *+H2B-RFP* flies (Fig 7H). This finding suggests that the vacuolization affects the glial cell body without causing cell loss. This finding is consistent with our EM results that demonstrated the nuclei in the vacuolated glia are intact (Fig 2E). A defect in lipid metabolism homeostasis can be involved in neuronal or glial degeneration [67]. However, we found no apparent change in lipid accumulation in the *repo* ^*ts*^ *>Shi* ^*ts1*^ lamina (S3B Fig compared with S3A Fig).

**Fig 8:**
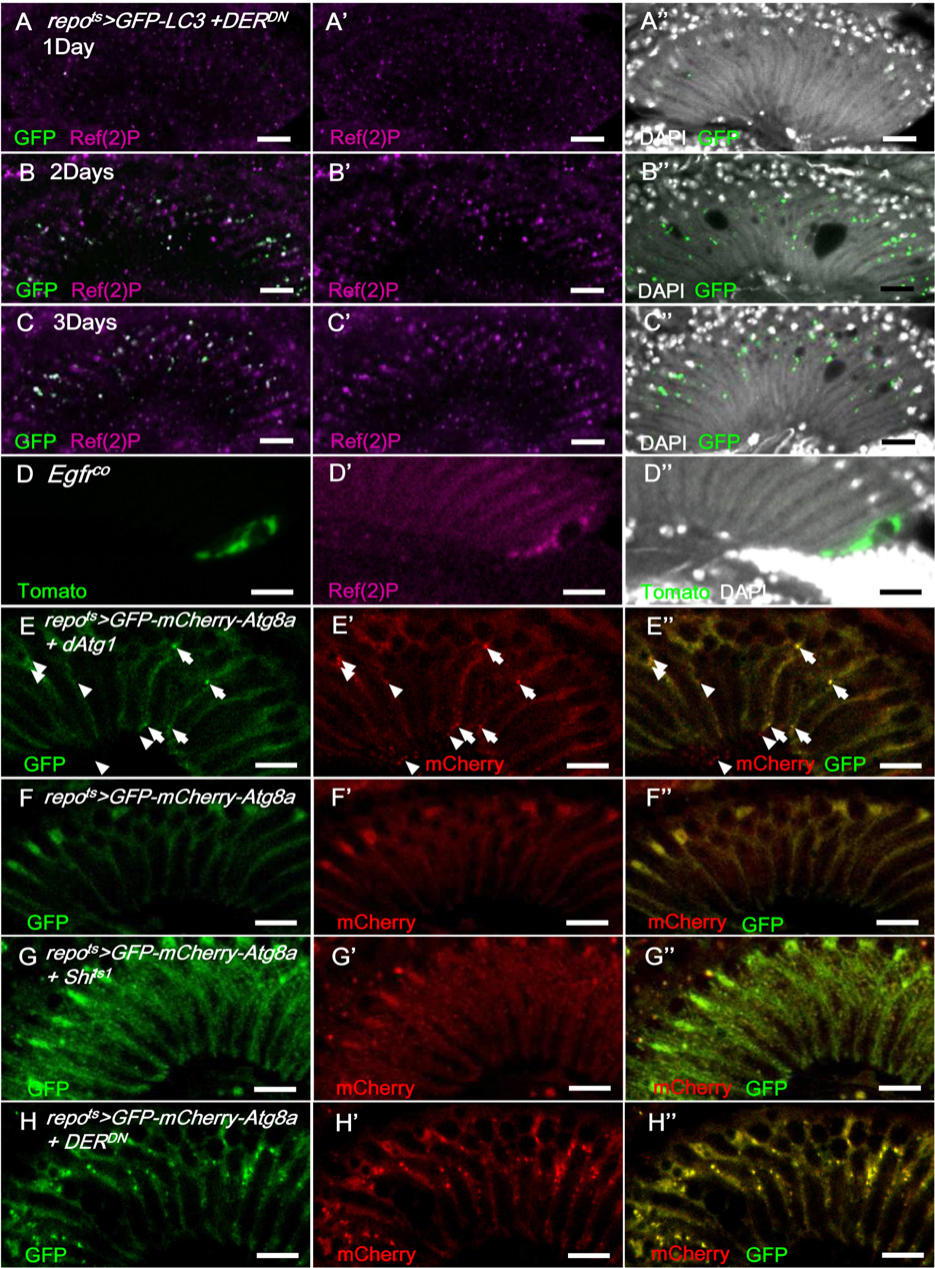
EGFR signaling is required for autophagosome-lysosomal trafficking. In *repo* ^*ts*^ *>GFP-LC3+DER* ^*DN*^ shifted to 28°C, the GFP signal (green) was weak on day 1 (A-A”) and progressively increased on days 2 (B-B”) and 3 (C-C”) and colocalized with Ref(2)P (magenta). (D) Ref(2)P (magenta) accumulated within the *Egfr* ^*co*^ mutant MARCM clone (green). The double-tagged GFP-mCherry-Atg8a is used to distinguish the autophagosomes (GFP and mCherry, yellow) and autolysosomes (mCherry, red) in autophagic flux. (E) In *repo* ^*ts*^ *>GFP-mCherry-Atg8a+dAtg1*, autophagosomes (arrowhead) and autolysosomes (arrow) were induced in the glia with normal autophagic flux. (F) *repo* ^*ts*^ *>GFP-mCherry-Atg8a*. (G) *repo* ^*ts*^ *>GFP-mCherry-Atg8a+Shi* ^*ts1*^. Epithelial glial nuclei are indicated (arrow). (H) *repo* ^*ts*^ *>GFP-mCherry-Atg8a+DER* ^*DN*^. The adults were shifted to 28°C for 3 days. GFP: green (E-H); mCherry: red (E’-H’); merge (E”-H”). Scale bar: 10 μm.

Autophagy-like vesicles that encapsulated bulk cargo and organelles were identified in the glia of *repo>Shi* ^*ts1*^ and *repo>DER* ^*DN*^ flies (Fig 2D’ and 2E’). We subsequently assessed the levels of the autophagy markers GFP-LC3 [68] and Ref(2)P, the *Drosophila* ortholog of p62 [69]. Atg8/LC3 requires activation via proteolytic cleavage by Atg4 and is subsequently conjugated to phosphatidylethanolamine by Atg7 and Atg3. Therefore, Atg8/LC3 overexpression in the fly does not enhance autophagy [70] and is generally used as an inconspicuous marker of autophagy. In the *repo* ^*ts*^ *>GFP-LC3+DER* ^*DN*^ adult flies shifted to 28°C, the GFP-LC3 puncta became detectable on day 2 (Fig 8B compared with Fig 8A). Ref(2)P typically binds to LC3 and is degraded in the autolysosomes; however, it accumulates in the presence of autophagosome-lysosomal trafficking defects and neurodegenerative diseases [71–74]. In the *repo* ^*ts*^ *>DER* ^*DN*^ flies, the Ref(2)P signal was weak on day 1 (Fig 8A); however, it accumulated in the glia and colocalized with the LC3-GFP puncta between days 2 and 3 (Fig 8B and 8C). The accumulation of Ref(2)P was also identified cell-autonomously in the *Egfr* ^*co*^ mutant glial clone, which suggests that the *repo>DER* ^*DN*^ effect is a result of the loss of EGFR signaling rather than an effect of DER^DN^ (Fig 8D). We further examined the specific compartment in which the autophagosomal cargo accumulated. The double-tagged GFP-mCherry-Atg8a contains mCherry, which is resistant to the low pH in the lysosome, and GFP, which is quenched in the lysosome. Therefore, this tag can be used to distinguish the autophagosomes (GFP and mCherry, yellow) from the autolysosomes (mCherry, red) during autophagic flux (Fig 8E) [75, 76]. In the *repo* ^*ts*^ *>Shi* ^*ts1*^ and *repo* ^*ts*^ *>DER* ^*DN*^ flies, the induced puncta signal predominately appeared in the autophagosomes versus the autolysosomes (Fig 8G and 8H compared with 8F). These results indicate that EGFR signaling in the glia promotes the fusion of autophagosomes to lysosomes. The absence of EGFR signaling caused a failure in Atg8 and Ref(2)P degradation and resulted in their accumulation in the autophagosomes.

These results suggest that autophagy may contribute to glial vacuolization. Autophagy gene *dAtg1* expression in the glia (*repo* ^*ts*^ *>dAtg1*) induced a similar lamina vacuolization phenotype compared with the *repo* ^*ts*^ *>Shi* ^*ts1*^ flies (S4A Fig). When *repo* ^*ts*^ *>DER* ^*DN*^ adults were treated with the autophagy inhibitor 3-methyladenine (3-MA), the vacuolization was partially rescued (S4B Fig). The *repo* ^*ts*^ *>Shi* ^*ts1*^ and *repo* ^*ts*^ *>DER* ^*DN*^ vacuolization phenotypes, which were repressed by the coexpression of the autophagy induction blocker dTOR [77], were repressed by reducing Atg1 and Atg13 (S4C and S4D Fig), and enhanced by coexpressing an activated form of the autophagy-promoting S6K (S6K^STDETE^) (S4E and S4F Fig). A knockdown of the autophagy proteins Atg5, Atg7, and Atg12 alleviated the vacuolization phenotype of the *repo* ^*ts*^ *>Shi* ^*ts1*^ flies; however, it was not sufficient to rescue the stronger phenotype of the *repo* ^*ts*^ *>DER* ^*DN*^ flies (S4E and S4F Fig). These results indicate that autophagy is, at least in part, responsible for the glia vacuolization phenotype.

### EGFR signaling is required for trafficking to lysosomes

**Fig 9:**
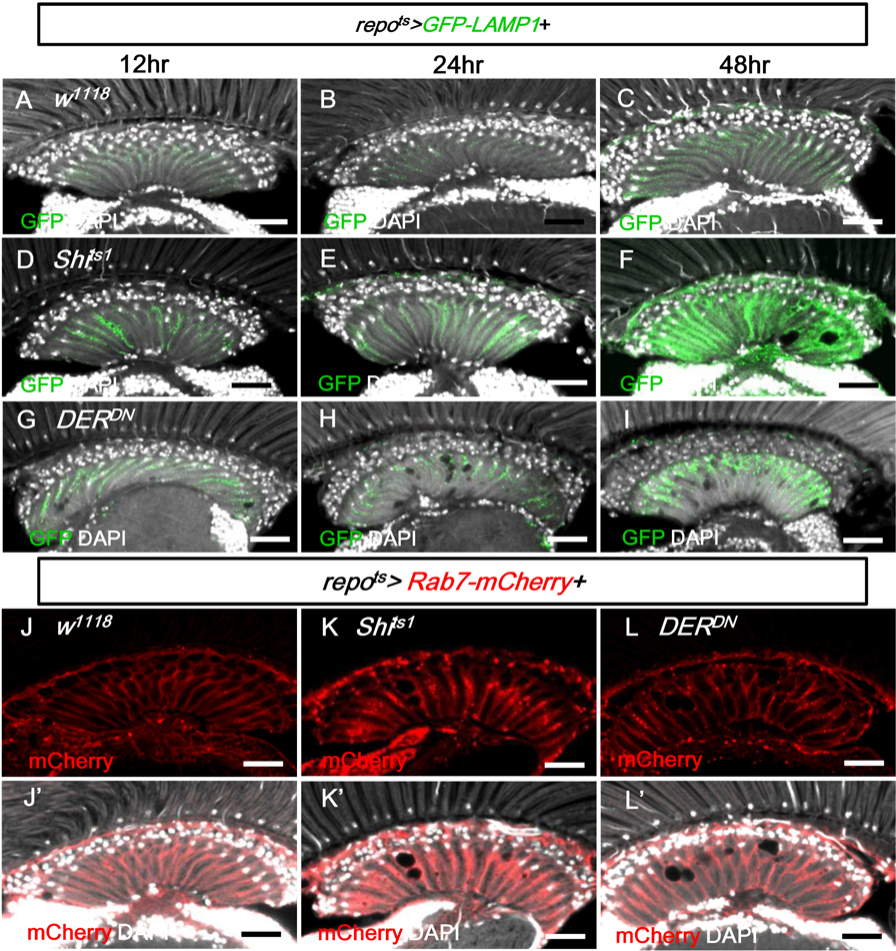
EGFR signaling is required for endo-lysosomal trafficking. (A-C) *repo* ^*ts*^ *>GFP-LAMP1*. (D-F) *repo* ^*ts*^ *>GFP-LAMP1+Shi* ^*ts1*^. (G-I) *repo* ^*ts*^ *>GFP-LAMP1+DER* ^*DN*^. Adults were incubated at 28°C for 12 h (A, D, G), 24 h (B, E, H), and 48 h (C, F, I), respectively. The GFP-LAMP1 signal (green) was induced at 12 h and progressively enhanced in (D-F) and (G-I). (J) *repo* ^*ts*^ *>Rab7-mCherry+H2B-RFP*. (K) *repo* ^*ts*^ *>Rab7-mCherry+Shi* ^*ts1*^. (L) *repo* ^*ts*^ *>Rab7-mCherry+DER* ^*DN*^. The adults were shifted to 28°C for 2 days. *Rab7-mCherry* puncta (red) were increased in (K, L). DAPI: nuclei (white in J’-L’). Scale bar: 20 μm.

We next examined the effect on GFP-LAMP1, which is targeted to the membrane of the late endosome/lysosome and subsequently degraded in the mature lysosomes [78, 79]. When GFP-LAMP1 was expressed in the glia (*repo* ^*ts*^ *>GFP-LAMP1*), the GFP signal was weak (Fig 9A–9C). In the *repo* ^*ts*^ *>Shi* ^*ts1*^ and *repo* ^*ts*^ *>DER* ^*DN*^ flies, the GFP-LAMP1 signal was significantly increased in the lamina after only 12 h at 28°C and was strongly accumulated on day 2 (Fig 9D–9I). The increased GFP-LAMP1 accumulation identified in the *repo* ^*ts*^ *>Shi* ^*ts1*^ flies was reduced when the EGFR signaling was enhanced using a gain-of-function allele *rl* ^*Sem*^ */+* and was enhanced when the doses of the EGFR ligands Spi and Krn were reduced (S5D–S5G Fig). In all conditions, the severity of lamina vacuolization correlated with the GFP-LAMP1 intensity (Figs 4G, 4H, S5G, S5H). The early and strong accumulation of GFP-LAMP1 also suggests that the impairment of the lysosomal system may be the primary cause of the glia vacuolization.

In the *repo* ^*ts*^ *>Shi* ^*ts1*^ and *repo* ^*ts*^ *>DER* ^*DN*^ flies, the late endosome marker Rab7-mCherry also accumulated as puncta in the lamina (Fig 9K and 9L compared with 9J). Taken together with the accumulation of GFP-LC3, Ref(2)P and LAMP1-GFP, these results suggest that the trafficking or the fusion of the late endosome and autophagosome to the lysosome is blocked.

The accumulation of the autophagosomal proteins GFP-LC3 and Ref(2)P may be a result of a failure in lysosomal degradation or autophagosome-lysosomal trafficking. Feeding the *repo* ^*ts*^ *>Shi* ^*ts1*^ and *repo* ^*ts*^ *>DER* ^*DN*^ flies with chloroquine, which inhibits lysosomal acidification and degradation [80, 81], did not affect the LAMP1-GFP phenotype (S6 Fig). This result suggests that the GFP-LAMP1 accumulation could be because of a block at a step upstream of lysosomal degradation. In this case, a block downstream of lysosomal degradation would not affect the upstream blockage.

The overexpression of the apoptotic protein Hid did not induce GFP-LAMP1 accumulation, vacuolization, or autophagy accumulation in the glia (S5G and S2C and S2D Figs), which suggests that the lysosomal defect in the glia is not a response to apoptosis. While the overexpression of the autophagy gene dAtg1 in the glia caused lamina vacuolization (S4E Fig), it did not cause GFP-LAMP1 accumulation (S5C and S5G Fig), which suggests that autophagy is not induced upstream of the lysosomal defect. Because the autophagy marker GFP-LC3 was increased only 2 days after blocking EGFR signaling (Fig 8B), these results suggest that autophagy is a late event in glia vacuolization and may be a secondary response or independent of the lysosomal impairment.

**Fig 10:**
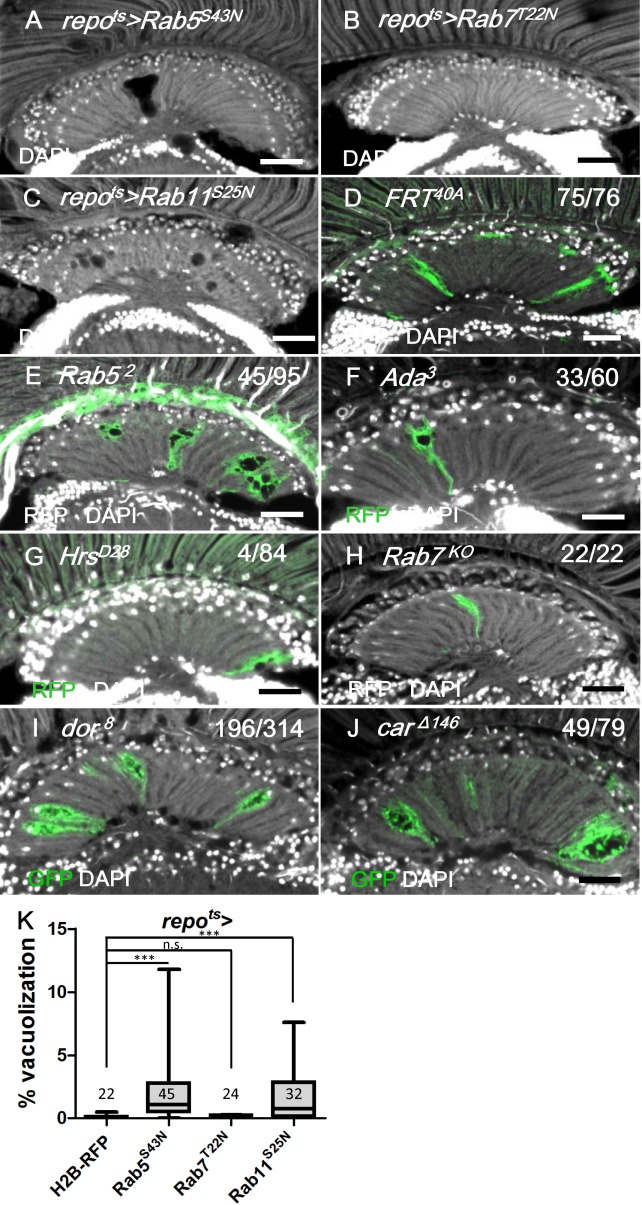
Defect in early endocytic steps and lysosomal trafficking caused lamina vacuolization. (A) *repo* ^*ts*^ *>Rab5* ^*S43N*^. (B) *repo* ^*ts*^ *>Rab7* ^*T22N*^. (C) *repo* ^*ts*^ *>Rab11* ^*S25N*^. (D-H) MARCM clones labeled by RFP or GFP (green) of control (D), *Rab5* ^*2*^ (E), *α-Ada* ^*3*^ (F), *Hrs* ^*D28*^ (G), *Rab7* ^*KO*^ (H), *dor* ^*8*^ (I) and *car* ^*Δ146*^ (J). The penetrance (number of samples with vacuole over the number of samples examined) is indicated in each panel. Adults of all genotypes were incubated at 28°C for 14 days. DAPI: nuclei (white in A-J). Scale bar: 20 μm. (K) The percentage of the vacuole area in (A-C) was summarized. Adults of these genotypes were incubated at 28°C for 12 days. *P*-values were calculated using Kruskal-Wallis with Dunn’s post-tests.

Considering that these results demonstrated that blocking an early step of the endocytic pathway in the *repo>shi* ^*ts1*^ flies caused vacuolization, we investigated other components of the vesicle trafficking pathways. Rab5 is required for the fusion of the endocytic vesicles with the early endosome [82]. The expression of a dominant-negative Rab5 (Rab5^S43N^) [83] in the adult glia (*repo* ^*ts*^ *>Rab5* ^*S43N*^) caused lamina vacuolization (Fig 10A and 10K) and enhanced the GFP-LAMP1 signal (S5A and S5G Fig) via similar effects as the phenotype observed in the *repo>Shi* ^*ts1*^ flies (Figs 4A and 9F). *Rab5* ^*2*^ mutant MARCM clones also exhibited lamina glia vacuolization (Fig 10E). α-Adaptin (α-*Ada*) is a subunit of the AP-2 complex, which is required for endocytosis [84]. Vacuoles could be identified in the lamina glia of the α-*Ada* ^*3*^ mutant clones (Fig 10F). These data suggest that the early steps of endocytosis, which involve Shi, Rab5 and Ada, are required for lamina glia maintenance. Activated EGFR is endocytosed and continues to signal from the early endosome [29]; thus, these results suggest that EGFR signaling from the early endosome is important to prevent vacuolization of the lamina glia.

We also examined other steps involved in vesicle trafficking. Hrs is required for the transition from the early endosome to the late endosomes or multivesicular bodies (MVB) [29]. The *Hrs* ^*D28*^ homozygous mutant clones did not exhibit vacuolization (Fig 10G). Rab7 is required for the docking of the early endosome to the late endosome, as well as the fusion of the late endosome and autophagosome with the lysosome [85, 86]. The expression of a dominant negative form of Rab7 (Rab7^T22N^) did not cause lamina vacuolization (Fig 10K) or GFP-LAMP1 accumulation (S5B and S5G Fig) [87]. Because the endolysosomal conversion was not affected by Rab7^T22N^, which suggested that this mutant could not be a dominant-negative form [88], a *Rab7* ^*KO*^ mutant clone was generated and did not exhibit vacuolization in the lamina (Fig 10H). Rab11 is required in recycling endosomes and promotes the fusion of late endosomes or MVBs with autophagosomes [89, 90]. The expression of the dominant-negative Rab11^S25N^ in the glia caused vacuolization [91] (Fig 10C and 10K), which indicates that either recycling endosomes or autophagosome maturation may also be involved in the maintenance of cell integrity. Our results suggest that the vesicle trafficking steps that involve Hrs and Rab7 are not required to prevent lamina glia vacuolization. This finding was consistent with the lack of EGFR signaling from the late endosomes [29].

**Fig 11:**
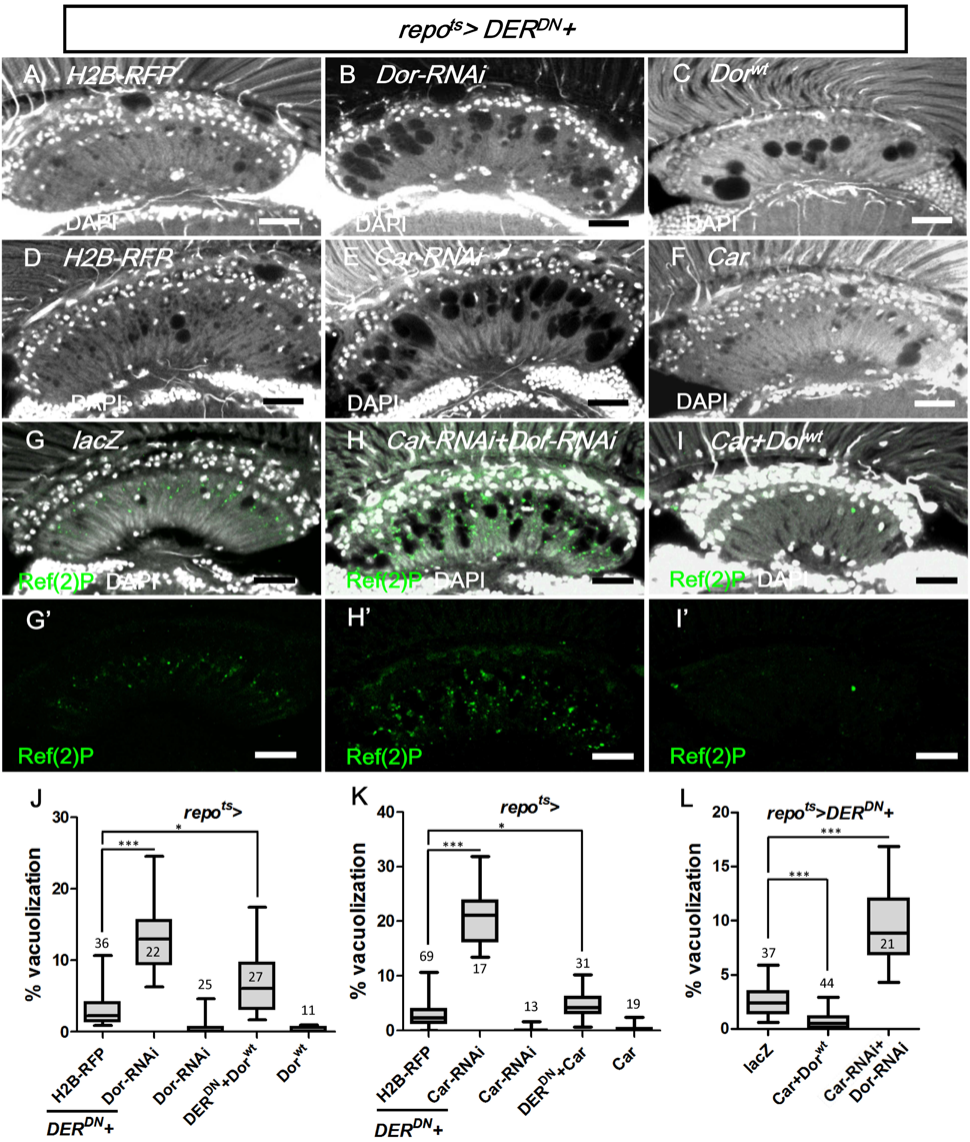
Vps-C complex components Dor and Car affected lamina glial vacuolization. *repo* ^*ts*^ *>DER* ^*DN*^ was coexpressed with (A) *H2B-RFP*, (B) *Dor* ^*wt*^, (C) *Dor-RNAi*, (D) *H2B-RFP*, (E) *Car*, (F) *Car-RNAi*, (G) *lacZ*, (H) *Car-RNAi+Dor-RNAi* and (I) *Car+Dor* ^*wt*^. The autophagy reporter Ref(2)P (green) was stained (G’-H’). Scale bar: 20 μm. (J, K, L) The percentages of the vacuole areas in the lamina of (A-C, D-F, G-I) were summarized, respectively. The adults were shifted to 28°C for 7 days. DAPI: nuclei (white in A-I). All *P*-values were calculated using Kruskal-Wallis tests with Dunn’s post-tests.

The class C vacuolar protein-sorting (Vps) complex plays a role in vesicle sorting and trafficking between different vesicular compartments. Deep orange (Dor) and Carnation (Car) are subunits of the Vps-C complex and are involved in the trafficking between late endosomes and lysosomes [78, 92, 93]. The depletion of Dor and Car in the fat body caused autophagosome accumulation [94, 95]. Therefore, we assessed whether *dor* and *car* were involved in glia vacuolization. We identified a high frequency of vacuolization in the *dor* or *car* mutant glial clones (Fig 10I and 10J). Although knockdown of Dor or Car alone in the glia did not cause vacuolization (Fig 11J and 11K), it enhanced lamina vacuolization in the *repo* ^*ts*^ *>DER* ^*DN*^ flies (Fig 11B, 11E, 11J, 11K compared with 11A and 11D). Surprisingly, glial vacuolization and GFP-LAMP1 accumulation in the *repo* ^*ts*^ *>DER* ^*DN*^ flies were also slightly enhanced by the coexpression of wild-type Dor or Car (Figs 11C and 11F and S7), although the expression of Dor or Car in the wild-type did not cause a defect (Fig 11J and 11K). Both a reduction and increase in the dosage of Dor or Car enhanced the *repo* ^*ts*^ *>DER* ^*DN*^ flies vacuolization phenotype; thus, these results suggest that a proper balance in the expression of the Vps-C complex components is essential for glia maintenance. The knockdown of both Car and Dor strongly enhanced lamina vacuolization and Ref(2)P accumulation in the *repo* ^*ts*^ *>DER* ^*DN*^ flies (Fig 11H and 11L compared with 11G). By coexpressing both Car and Dor in the *repo* ^*ts*^ *>DER* ^*DN*^ flies, both vacuolization and Ref(2)P accumulation were rescued (Fig 11I and 11L). Although we cannot exclude the possibility that EGFR signaling may act in parallel to the Vps-C complex, these genetic interactions suggest that EGFR signaling acts at a step upstream of Dor/Car in the promotion of the autophagic flux.

## Discussion

Presynaptic and postsynaptic neurons can mutually maintain the survival of their synaptic partners. During development, neurons can also provide gliotrophic factors to maintain glia survival. The majority of human neural degeneration exhibits a late onset and progresses over time; thus, the major concern is the maintenance of cell survival or function. For hereditary neural degenerations or genetically manipulated animal models of neural degeneration, it is typically difficult to separate the developmental effects from the true maintenance requirement in adults. Our experimental approach specifically bypassed the development and examined the events at the adult stage, which therefore addresses the maintenance of the adult visual system in a manner more relevant to human nervous system degeneration. Our results demonstrate for the first time that the adult photoreceptor neurons actively maintain the integrity of glia within their target field in the optic lamina.

We demonstrated that in the adult visual system, the R1-6 photoreceptors produce and transport the EGFR ligand Spi, and presumably Krn, to the axon termini in the optic lamina to act on the EGFR in the lamina epithelial and marginal glia to maintain integrity. Spi and Krn are the first gliotrophic factors demonstrated to act in the adult nervous system. Because of the advantages offered by the fly visual system, we were able to clearly define the source and recipient cell types for the gliotrophic signal.

Photoreceptors secrete gliotrophic factors most likely to maintain the functional integrity of their microenvironment and its synapses. The epithelial glia is involved in the reuptake of neurotransmitters from the synaptic cleft and their metabolism. In the absence of EGFR signaling, the lamina glia undergoes a progressive and irreversible vacuolization, which is accompanied by a defect in photoreceptor synaptic transmission. Interestingly, this degeneration is not because of apoptosis and does not involve cellular losses. This conclusion is based on the following observations: (1) there was no apparent loss of Repo^+^/DAPI^+^ nuclei number in the epithelial glia layer in the degenerating lamina, (2) there was no apoptotic signal (assessed by anti-activated caspase 3, TUNEL assay, and Apoliner) in the degenerating lamina, (3) the glia nuclei in the degenerating lamina appeared intact (assessed by Repo, DAPI staining and EM), (4) the coexpression of the anti-apoptotic P35 or Diap1 failed to rescue the phenotype, and (5) the *repo* ^*ts*^ *>hid* flies did exhibit lamina vacuolization or autophagy (GFP-Cherry-Atg8a). Thus, adult lamina glia degeneration represents a new type of cellular degeneration with the loss of cellular integrity and function, but without the loss of cell number.

Most studies have focused on neurons in these degenerative conditions. We now provide a model system in which the glial cells are the primary degenerating cells. It would be interesting and important to determine whether the gliotrophic maintenance is also at play when the nervous system is damaged by trauma or other pathological conditions, as demonstrated for the response to injury in the larval ventral nerve cord [96].

EGFR ligand-binding on the cell surface activates the receptor and results in the transduction of a signal into the nucleus. The ligand-bound receptor becomes internalized by endocytosis. Internalized EGFR can exhibit a sustained level of activation and signaling from the early endosome [28–30]. In our study, endocytosis is blocked in the *repo>Shi* ^*ts1*^ flies, which presumably results in more activated EGFR at the cell surface. This effect caused glia degeneration, which suggests that the cell surface EGFR signaling is not sufficient to maintain glial integrity. However, the *repo>Shi* ^*ts1*^ phenotype could be rescued by the coexpression of activated EGFR, which would remain on the cell surface because endocytosis is blocked by Shi^ts^. This rescue indicates that increased cell surface EGFR signaling can replace the missing EGFR signaling from the early endosomes. Therefore, EGFR signaling from the two compartments, namely, the cell surface and early endosome, are qualitatively the same and may only be different in terms of signaling intensity (S6 Fig).

EGFR can signal via multiple mechanisms [97]. The membrane-bound EGFR can signal via its tyrosine kinase activity through the Ras-Ref-MEK-MAPK, PI3K- Akt-mTOR, PLC-γ-PKC, and Jak2-STAT3 pathways. EGFR can also signal via kinase-independent mechanisms most likely through interactions with other proteins [97]. Our results demonstrated that in the lamina glia, EGFR signals through the MAPK pathway. Ligand-activated EGFR can also enter the cells and exert certain functions in the nucleus and mitochondria [97]. The nuclear transport of EGFR requires endocytosis [98, 99]. Whether the mitochondrial transport of EGFR requires endocytosis is controversial [100, 101]. The nuclear and mitochondrial transport of EGFR has not been reported in *Drosophila*. We demonstrated that the early endocytic steps that involved Shi, Rab5, and α-Ada were required to prevent lamina glia degeneration, which suggests that the internalized EGFR signals from the early endosome. However, we cannot exclude the possibility that EGFR signals from the nucleus or mitochondria because blocking endocytosis would also block the nuclear transport, and possibly the mitochondrial transport, of activated EGFR.

In mouse cortical astrocytes and *Drosophila* embryonic CNS glia, the absence of EGFR signaling leads to glia apoptosis [6, 12, 102]. Our findings demonstrate that in the adult lamina, the absence of EGFR signaling triggers a different type of cellular degeneration, which is independent of apoptosis. The same Spi signal from the same photoreceptors is transported to the lamina and exerts different functions in each developmental stage. Spi acts on the lamina neurons during the larval stage for the differentiation of cartridge neurons [32], whereas it acts on the lamina glia in the adult for their maintenance.

There is no report that links EGFR signaling and autophagy in *Drosophila*. Our results suggest that the vacuolization is, at least in part, a result of autophagy. In cancer treatment with anti-EGFR antibodies and small molecule drugs that inhibit EGFR tyrosine kinase activity, autophagy is often induced [42]. This finding suggests that EGFR signaling can inhibit autophagy in the lamina glia. The mammalian EGFR can bind directly to the autophagy regulator Beclin-1 and inhibit autophagy [41]. It is unknown whether, in the fly, EGFR can also bind to and phosphorylate Atg6, the *Drosophila* Beclin-1 homolog. EGFR can also prevent autophagy via interaction with the sodium/glucose cotransporter 1 (SGLT1) in a kinase-independent manner to maintain the intracellular glucose level [103]. It is unknown whether a similar mechanism also operates in *Drosophila*. Our findings may be the first to link EGFR signaling to autophagy in *Drosophila*.

Blocking EGFR signaling in the glia caused several phenotypes. The accumulation of GFP-LAMP1 occurred 12 h after shifting to the non-permissive temperature. The ERG was normal on day 1; however, the ON/OFF transients were completely absent on day 3. The lamina vacuoles were noticeable on day 2 and became progressively more apparent. The autophagy marker GFP-LC3 increased on day 2. Because the accumulation of GFP-LAMP1 was the earliest and strongest effect, we suppose that this finding reflects the primary cause of the degeneration. Our results suggest that EGFR signaling is required for proper vesicle trafficking from the late endosome and autophagosome to the lysosome (S6 Fig). A failure at the fusion step of the late endosome or autophagosome to the lysosome caused the accumulation of autophagosomes and increased GFP-LC3 in the fly [92, 94, 95, 104], as well as in certain mammalian lysosomal storage diseases [105, 106]. The accumulation of autophagosomes may cause cellular degeneration perhaps because of the accumulation of certain proteins, typically destined for degradation, that become toxic to the cell and trigger autophagy [107]. Although we propose that the autophagy is a secondary cause of the failure in the autophagosome-lysosome fusion, we do not exclude the possibility that the loss of EGFR signaling could independently enhance autophagy. Our findings are the first study to link EGFR signaling with the trafficking from the late endosome and autophagosome to the lysosome.

EGFR signaling is increased in many cancers. Fifty to sixty percent of primary glioblastoma tumors exhibit increased EGFR signaling [108]. The EGFR signaling pathway has been a major therapeutic target for various types of cancer, including glioblastoma [109, 110]. The level of EGFR signaling must be well balanced because too much signaling can lead to oncogenic growth, whereas too little signaling may lead to glia degeneration, as demonstrated by our study. Therefore, our study highlights the caution needed in the therapeutic treatments that act via a reduction of EGFR signaling.

## Materials and Methods

### Fly stocks

Fly culture and crosses were performed according to standard procedures at 25°C unless otherwise noted. The fly stocks (*repo-Gal4*, *GMR-Gal4*, *point* ^*1277*^ *-lacZ*, *longGMR-Gal4*, *ey* ^*3*.*5*^ *-FLP*, *tubGAL80* ^*ts*^, *UAS-GFP*.*nls*, *UAS-lacZ*, *UASp-GFP-mCherry-Atg8a*, *UAS-Apoliner*, *UAS-DsRed*, *UAS-Hid*, *FRT* ^*19A*^ *tubP-GAL80 hs-FLP; UAS-mCD8-GFP*, *FRT* ^*G13*^ *tubGal80*, *FRT* ^*G13*^ *UAS-GFP*, *FRT* ^*80B*^ *tubGAL80*, *FRT* ^*42D*^ *tubGAL80*, *FRT* ^*40A*^ *tub-GAL80*, *UAS-Rab5* ^*S43N*^, *UAS-Rab7* ^*T22N*^, *UAS-Rab11* ^*S25N*^, *UAS-dTor* ^*WT*^, *UAS-S6K* ^*STDETE*^, *GMR-wIR* and *rdgC* ^*306*^) were obtained from the Bloomington Stock Center. The *rl* ^*Sem*^ was obtained from the Drosophila Genetic Resource Center. The *UAS-Spitz-RNAi* (KK103817) and *UAS-Keren-RNAi* (GD27110) were obtained from the Vienna Drosophila Research Center. The *UAS-Dor-RNAi* (3093R-4) and *UAS-Car-RNAi* (12230R-1) were obtained from the NIG-FLY. The *Rh1-GAL4 UAS-lacZ* was provided by Larry Zipursky. The *UAS-mCherry-Rab7* was provided by Jui-Chou Hsu. The *repo-GAL4*,*UAS-mRFP* was provided by Yuh Nung Jan, and the *UAS-P35* was provided by Bruce Hay. The following stocks were provided by the original authors: *Ln-GAL4* [111], *repo-FLP* [112], *repo-GAL4 UAS-CD4-mtdTomato* [113], *repo-GAL80* [114], *UAS-H2B-RFP* [115], *UAS-Shi* ^*ts1*^ [19], *UAS-Egfr* ^*λtop4*.*2*^ [116], *UAS-DER* ^*DN*^ [117], *UAS-Rl* ^*Sem*^ [118], *UAS-mSpiGFP* [56], *UAS-iRhom* [58], *UAS-dAtg1 and UAS-Atg1-RNAi* [119], *UAS-Atg5-RANi*, *UAS-Atg7-RNAi* and *UAS-Atg12-RNAi* [77], *Egfr* ^*co*^ [120], *spi* ^*OE92*^ [121], *Krn* ^*27-7-B*^ [122], *rho1* ^*7M43*^ *ru* ^*1*^ [123], *dor* ^*8*^ and *UAS-Dor* ^*wt*^ [124], *car* ^*Δ146*^, *UAS-GFP-LAMP1 and UAS-Car* [92], *atg13* ^*Δ81*^ [125], *Rab5* ^*2*^ [82], *Rab7* ^*KO*^ [126], *α-Adaptin* ^*3*^ [84], *Hrs* ^*D28*^ [29], *UAS-GFP-LC3* [68], *repo-FLP repo-GAL4 UAS-actGFP; FRT* ^*82B*^ *tubGAL80* [112].

The *repo-Gal4* and *tubGAL80* ^*ts*^ were recombined into *repo-GAL4 tubGAL80* ^*ts*^ (*repo* ^*ts*^ *-GAL4*) on the third chromosome. The recombinant lines were selected by crossing with *UAS-Hid*. The *repo>Hid* is larva-lethal at room temperature; however, it is viable with *tubGal80* ^*ts*^. The recombinant of *repo-GAL4 UAS-Shi* ^*ts1*^ was selected by the lethality feature at 30°C for 7 days.

The genotypes for the MARCM clone generation were as follows: *FRT* ^*42D*^ *tubGAL80/FRT* ^*42D*^ *Egfr* ^*co*^ *; repo-GAL4 UAS-mtdTomato/repo-FLP*, *hs-FLP/+*; *FRT* ^*G13*^ *tub-Gal80/FRT* ^*G13*^ *UAS-mCD8GFP*; *repo-GAL4/UAS-Shi* ^*ts1*^, *FRT* ^*19A*^ *dor* ^*8*^ */FRT* ^*19A*^ *tubGAL80 hs-FLP; UAS-mCD8GFP; repoGal4/+*, *FRT* ^*19A*^ *car* ^*Δ146*^ */FRT* ^*19A*^ *tubGAL80 hs-FLP; UAS-mCD8GFP; repoGal4/+*. Forty-eight h after egg laying, the animals were heat-shocked for 90 min at 37°C.

The whole eye *rho* ^*7M43*^ *ru* ^*1*^ double mutant clones were generated from *ey* ^*3*.*5*^ *-FLP/UAS-DsRed; GMR-GAL4/+ FRT* ^*80B*^ *rho* ^*7M43*^ *ru* ^*1*^ */FRT* ^*80B*^ *tubGAL80* for 12 days.

### Conditional inactivation of Shi^ts1^ and GAL80^ts^

The crosses and flies were maintained at 17 or 21°C (permissive temperature) until adult eclosion. The adults (3–7 days old) were shifted to a restrictive temperature (28 or 29°C) to enable transgene expression for the indicated time.

### Hematoxylin & eosin (H&E)-stained paraffin sections

The fixed fly heads were dehydrated in series of ethanol/ddH_2_O steps, embedded in wax, and sectioned in paraffin blocks at 5–7 μm thickness. The sectioned head slides were deparaffinized with xylene and rehydrated in a series of ethanol/ddH_2_O. The slides were immersed in hematoxylin (Thermo Fisher Scientific) for 2 min and eosin (Thermo Fisher Scientific) for 5 min. Permount was added on the slides, which were imaged on a Zeiss AxioImager-Z1 microscope equipped with Plan Apo 20X DIC II and Plan Apo 40X DIC III immersion objectives.

### Immunohistochemistry and confocal microscopy

*GMR-wIR* is a *White-RNAi* driven by a *GMR* enhancer to reduce the autofluorescence from the retinal pigments. For cryosectioning, adult flies were fixed in 4% paraformaldehyde for 3 h at room temperature. The fly heads with proboscis were removed and incubated in 1x PBS that contained 25% sucrose at 4°C for 24 h and embedded in OCT compound (Tissue-Tek, Sakura). The solidified samples were sliced at a 100-μm thickness using a Leica LX2501 cryostat. The slices were incubated with the following primary antibodies: mouse anti-Repo (1:100), rat anti-Spitz (1:50) (Developmental Studies Hybridoma Bank), rabbit anti-β-Gal (1:500; Cappel), rabbit anti-Cleaved Caspase-3 (Asp175, 1:200, Cell Signaling), rabbit anti-full length Ref(2)P (1:300, a gift from Tor Erik Rusten), and guinea pig anti-Black (1:500, a gift from Bernhard Hovemann) [50]. The fluorescent secondary antibodies (1:200) were obtained from Jackson ImmunoResearch. DAPI (25 ng/ml, Sigma) was used to stain the DNA and tissue background. Immunolabeled slices were mounted in FocusClear (CelExplorer Labs) and imaged on a Zeiss LSM 510 Meta confocal microscope.

### Quantitative analysis

The severity of glial vacuolization in the lamina was quantified by outlining the vacuoles in the lamina. The area of the vacuole and lamina of each brain hemisphere was scored using Metamorph software (Molecular Devices). The measurement of GFP-LAMP1 fluorescence by image analysis generates intensity values that range from 1 to 255 using Metamorph software. The intensity of the collected images was assessed below the saturation level. The GFP intensity of each pixel in the lamina neuropile that was greater than the lower threshold (intensity value ≥25), as defined by the background, was averaged and expressed as the percentage of the mean values of the control genotype. For counting glial cell numbers, we used only females to avoid the differences in body size and sexual dimorphism in the brain. The lamina of 4°C cold-shocked adults were dissected, fixed in 4% paraformaldehyde at 4°C for 30 min, and imaged by Z-stacks of confocal images. The number of epithelial glial nuclei was examined by manually counting the nuclear RFP in the epithelial layer using Metamorph software. All data are presented as the means ± sem. The *P*-values of the multiple comparisons were obtained by one-way ANOVA for the normally distributed data and Kruskal-Wallis tests for the non-normally distributed data. The *P*-values of the two data sets were tested by unpaired Student *t*-tests for the normally distributed data and Mann—Whitney tests for the non-normally distributed data using GraphPad Prism software v5. Values of *P*<0.05 compared with the control group were considered statistically significant. *P<0.05, **P<0.01, ***P<0.001. n.s.: not significant. The N is indicated in the figures.

### TUNEL assay

The *In Situ* Cell Death Detection Kit (TMR red) was performed according to the user manual (Roche).

### Transmission electron microscopy (TEM)

Adult head sections for TEM were prepared as previously described [127].

### Drug treatment

Adult flies (3–7 days old) were pretreated with 5 mM of 3-Methyladenine (3-MA) or 1 mg/ml of Chloroquine (CQ) in 2% sucrose on tissue papers for 1 day at 17°C, followed by a temperature shift to 29°C for 4 and 2 days, respectively. During the incubation, the papers were kept moist and replaced once every 2 days.

### Electroretinogram (ERG)

Seven to eight adults of each genotype at the indicated age were placed in yellow tips, which were fixed by nail oil on the tip and left eye. The recording electrode touched on the surface of the right eye, and the ground electrode was on the head capsule. The flies were adapted in the dark for 30 s and stimulated by a 1-s 5000 Lux light pulse (Apex Monochromator Illuminator, 150 W Xenon Arc, Newport). The electrophysiological data were recorded via a microelectrode amplifier (Axonclamp 900A, Molecular Devices). The results were acquired using a data acquisition system (Digidata1440A, Molecular Devices) and analyzed using pClamp 10 software (Molecular Devices).

### Oil Red O staining

Cryosectioned fly heads were post-fixed in formal calcium (0.01 mg/ml CaCl_2_ in 4% paraformaldehyde pH 4.0) for 1 h and rinsed in deionized H_2_O and 50% isopropanol for 5 min. The slides were stained in an Oil Red O working solution (3 mg/ml Oil Red O in 60% isopropanol) for 6 min and rinsed in deionized H_2_O and 50% isopropanol for several seconds. The slides were stained by hematoxylin for 3 min (for nuclei staining), and the images were captured on an AxioImager-Z1 microscope (Zeiss) equipped with Plan Apo 20X DIC II and Plan Apo 40X DIC III immersion objectives.

## Supporting Information

S1 Fig *repo>Shi* ^*ts1*^ flies exhibited defective ERG. Electroretinogram (ERG) in response to a one second pulse of light in (A) *repo>Shi* ^*ts1*^ adults incubated at 21°C for 6 days, (B) 29°C for 1 day, (C) 29°C for 3 days, and (D) 29°C for 6 days. The On and OFF transients were progressively lost in the *repo>Shi* ^*ts1*^.
(EPS)

S2 Fig Lamina glial vacuolization and autophagy were not induced by apoptosis. (A) A few apoptotic signals (arrowhead) in the glia of *GMR-wIR; repo* ^*ts*^ *>Hid*. TUNEL (red) in (A). TUNEL (red) and Repo (green) in (A’). Active Caspase-3 (magenta) in (A”). Active Caspase-3 (magenta) and Repo (green) in (A”‘). The adults were shifted to 28°C for 5 days. Scale bar: 20 μm. (B) DNase treated *GMR-wIR; repo* ^*ts*^ *>lacZ* was used as a positive control for the TUNEL assay (red). TUNEL and Repo (green) in (B’) Scale bar: 20 μm. (C) Percentage of vacuole area in the lamina of (A). The *P*-values were calculated using unpaired Student’s *t-*tests. (D) Induction of apoptosis in *repo* ^*ts*^ *>GFP-mCherry-Atg8a+Hid* did not activate autophagy. The adults were shifted to 28°C for 5 days. GFP (green, D), mCherry (red, D’), merge (yellow, D”). Scale bar: 10 μm.
(EPS)

S3 Fig No lipid accumulation in the degenerating lamina. Oil Red staining of (A) *repo>shi* ^*ts1*^ at 17°C for 7 days and (B) *repo>shi* ^*ts1*^ shifted to 29°C for 7 days. Oil Red O-labeled fat cells near the optic lobe (red), which served as a positive control for the staining. The cell nuclei were labeled by hematoxylin (blue).
(EPS)

S4 Fig Autophagy contributed to lamina glial degeneration. (A) The percentage of the vacuole area in the lamina in *repo* ^*ts*^ *>dAtg1* shifted to 28°C for 5 days exhibited lamina vacuolization. (B) Incubation of *repo* ^*ts*^ *>DER* ^*DN*^ adults with the autophagy inhibitor 3-methyladenine (3-MA) in a 2% sucrose solution. The adults were preincubated with 3-MA for 1 day at 17°C and then shifted to 29°C for 4 days with 3-MA. The percentage of the vacuole area in the lamina is shown. (C) *repo* ^*ts*^ *>Shi* ^*ts1*^ and (D) *repo* ^*ts*^ *>DER* ^*DN*^ adults exhibited reduced vacuolization when Atg1 and Atg13 were reduced in *Atg1-IR*, *atg13* ^*Δ81*^ */+*. The *P*-values in (A), (C) and (D) were calculated using unpaired Student’s *t*-tests. The *P*-values in (B) were calculated using Mann-Whitney tests. The percentage of the vacuole area in the lamina of *repo* ^*ts*^ *>Shi* ^*ts1*^ (E) and *repo* ^*ts*^ *>DER* ^*DN*^ (F) when combined with the coexpression of dTOR^WT^, S6K^STDETE^, Atg5-IR, Atg7-IR and Atg12-IR, respectively. (C, E) and (D, F) were cultured at 28°C for 12 and 7 days, respectively. The *P*-values in (E, F) were calculated using one-way ANOVAs with Dunnett’s post-tests.
(EPS)

S5 Fig Intensity of early-accumulated GFP-LAMP1 positively correlated with lamina vacuolization severity. (A) *repo* ^*ts*^ *>GFP-LAMP1+Rab5* ^*S43N*^. (B) *repo* ^*ts*^ *>GFP-LAMP1+Rab7* ^*T22N*^. (C) *repo* ^*ts*^ *>GFP-LAMP1+dAtg1*. (D) *repo* ^*ts*^ *>GFP-LAMP1+Shi* ^*ts1*^. (E) *repo* ^*ts*^ *>GFP-LAMP1+Shi* ^*ts*^, *rl* ^*Sem*^ */+*. (F) *repo* ^*ts*^ *>GFP-LAMP1+Shi* ^*ts*^, *spi* ^*OE92*^ */+; Krn* ^*27-7-B*^ */+*. DAPI: nuclei (white). Scale bar: 20 μm. Adults were shifted to 28°C for 2 days. The GFP intensity in C, F, I, and J-L was normalized and is summarized in (G). The GFP intensity in D-F was normalized and is summarized in (H). All *P*-values were calculated using Kruskal-Wallis with Dunn’s post-tests.
(EPS)

S6 Fig Blockade of lysosomal degradation did not affect the GFP-LAMP1 accumulation because of the lack of EGFR signaling. The GFP intensities of *repo* ^*ts*^ *>GFP-LAMP1+Shi* ^*ts1*^ or *DER* ^*DN*^ adults treated with or without 1 mg/ml of chloroquine (CQ) were normalized and summarized. Adults were preincubated with CQ for 1 day at 17°C and then continued for 2 days at 28°C. The *P*-value was calculated using a Mann-Whitney test.
(EPS)

S7 Fig Vps C components do not affect the GFP-LAMP1 accumulation caused by EGFR signaling blockade. The normalized GFP intensity of *repo* ^*ts*^ *>GFP-LAMP1+DER* ^*DN*^ combined with the knockdown or overexpression of the Vps-C complex genes, Dor and Car, are summarized. *P*-values were calculated using Kruskal-Wallis with Dunn’s post-tests.
(EPS)

S8 Fig Model of the EGFR signaling-mediated mechanisms of glia maintenance. The ligand-activated EGFR is internalized into the early endosome in a process dependent on Shi, a-Ada and Rab5. EGFR continues to signal from the early endosome. The intensity of EGFR signaling from the early endosome is stronger than the signaling from the cell surface. EGFR signaling, via an unknown mechanism, is required for the fusion of the late endosome and autophagosome to the lysosome. Therefore, EGFR promotes its own degradation by forming a negative feedback loop, which may occur to prevent an over-activation of the EGFR pathway. In the absence of EGFR signaling, the autophagic flux is blocked, which results in the accumulation of proteins typically destined to be degraded, including GFP-LC3, Ref(2)P and LAMP1-GFP in the autophagosomes and Rab7 in the late endosome. The abnormal accumulation may be toxic to the cells and cause cellular vacuolization and dysfunction. In the absence of Rab7, the abnormal accumulation may not occur because Rab7 is also required for the trafficking in the early endosome to the late endosome.
(EPS)
